# An Advanced Whale Optimization Algorithm for Grayscale Image Enhancement

**DOI:** 10.3390/biomimetics9120760

**Published:** 2024-12-14

**Authors:** Yibo Han, Pei Hu, Zihan Su, Lu Liu, John Panneerselvam

**Affiliations:** 1 School of Computer and Software, Nanyang Institute of Technology, Nanyang 473004, China; 2 Department of Mathematics & Statistics, Hong Kong Baptist University, Hong Kong 519087, China; 3 Department of Informatics, University of Leicester, Leicester LE1 7RH, UK

**Keywords:** image enhancement, grayscale images, whale optimization algorithm

## Abstract

Image enhancement is an important step in image processing to improve contrast and information quality. Intelligent enhancement algorithms are gaining popularity due to the limitations of traditional methods. This paper utilizes a transformation function to enhance the global and local information of grayscale images, but the parameters of this function can produce significant changes in the processed images. To address this, the whale optimization algorithm (WOA) is employed for parameter optimization. New equations are incorporated into WOA to improve its global optimization capability, and exemplars and advanced spiral updates improve the convergence of the algorithm. Its performance is validated on four different types of images. The algorithm not only outperforms comparison algorithms in the objective function but also excels in other image enhancement metrics, including peak signal-to-noise ratio (PSNR), feature similarity index (FSIM), structural similarity index (SSIM), and patch-based contrast quality index (PCQI). It is superior to the comparison algorithms in 11, 6, 11, 13, and 7 images in these metrics, respectively. The results demonstrate that the algorithm is suitable for image enhancement both subjectively and statistically.

## 1. Introduction

Images are a primary source of human perception of the world and a key method for receiving and transmitting digital information [1,2]. Due to the influence of sensors, weather, and other factors, images are susceptible to issues such as clarity, contrast, and brightness degradation, which seriously affect subsequent information extraction and pattern recognition [3]. Therefore, image enhancement is essential for improving image quality.

Image enhancement is an important factor in the preliminary stage of image processing as it emphasizes the region of interest (ROI) within images [4]. Currently, it is widely used in medicine, agriculture, marine research, and more [5]. Given the importance of image enhancement, researchers have developed many algorithms. These methods are currently divided into spatial domain enhancement and transform domain enhancement. In the spatial domain, contrast enhancement utilizes nonlinear functions for gray-level transformation, including logarithmic transformations, gamma functions, histogram-based techniques, and nonlinear quadratic filtering.

Histogram equalization (HE) is a common spatial domain method for improving image quality because of its effectiveness and simplicity [6]. HE maps the grayscale levels of an image to new levels based on the image’s histogram and cumulative distribution function (CDF). However, a major drawback of traditional HE is its tendency to modify image brightness, leading to unwanted visual artifacts in the processed image. Various extensions of HE have been proposed to address these issues, but their effectiveness has been constrained [7].

Besides HE-based methods, image enhancement can also be performed using other techniques such as contrast stretching, low-pass and high-pass filters, Retinex models, and swift algorithms [8]. These techniques often fail to produce satisfactory results for a wide range of low-contrast images. In this regard, metaheuristic algorithms have received extensive attention due to their randomness and robustness [9,10,11,12], and particle swarm optimization (PSO), genetic algorithm (GA), differential evolution (DE), and other algorithms have been employed to automatically enhance image contrast [13,14,15].

Image enhancement algorithms, including traditional optimization methods and metaheuristic-based approaches, iteratively adjust parameters to explore the solution space and maximize the objective function. However, without robust exploration strategies, these algorithms may prematurely converge to suboptimal solutions. In this paper, we improve the whale optimization algorithm (WOA) for image enhancement, and the main contributions are as follows:

1. One limitation of the traditional WOA is its slow convergence rate, especially in high-dimensional or complex optimization tasks. The modifications introduced to the whale optimization algorithm (AWOA), including advanced search strategies and population update, significantly enhance convergence speed without compromising the quality of solutions in grayscale image enhancement tasks.
2. A prevalent challenge in WOA is achieving an effective balance between exploration and exploitation. We utilize novel exemplars and advanced spiral updates to strengthen this balance, and the algorithm can effectively explore new regions of the solution space while fine-tuning existing solutions.
3. The proposed method outperforms existing algorithms in peak signal-to-noise ratio (PSNR), feature similarity index (FSIM), structural similarity index (SSIM), patch-based contrast quality index (PCQI), and convergence curves.

The main structure of this paper is as follows: Section 2 introduces related works on image enhancement, and Section 3 describes the image enhancement model based on WOA. The image enhancement experiments and analysis are presented in Section 4, and Section 5 provides a summary and outlook of the work.

## 2. Related Works

Image enhancement techniques improve the appearance of images to achieve better visual interpretation. They also boost the performance of subsequent tasks such as computer vision, image segmentation, image processing, and object detection and tracking. This section provides a brief overview of image enhancement research based on metaheuristic algorithms.

Image enhancement aims to enhance visual quality by increasing the contrast of images that may have been distorted or diminished during capture. HE is the most commonly used method for this task. However, the exhaustive methods used in HE are algorithmically complex. Ahmed et al. used grayscale mapping techniques to transform images into solutions for an optimization problem and employed barnacles mating optimizer (BMO) to find the optimal solution for this problem [16]. Woldamanuel provided an improved HE method using the water cycle algorithm (WCA) to enhance image contrast while preserving brightness [17]. The experimental results indicate that the proposed technique is superior to traditional methods in terms of objective function values and perceived visual quality. Kamoona et al. introduced a novel enhanced cuckoo search (ECS) algorithm for image enhancement [18]. In order to address problems caused by local/global enhancement transformations that strengthen edges and cause image distortion, they suggest a new range for searching for transformation parameters that produce better-enhanced images. Qin et al. proposed an improved PSO algorithm and applied it to infrared image enhancement [15]. They develop a new exponential central symmetric inertia weight function and introduce a mechanism for escaping local optima. Additionally, they propose a new method for image enhancement by combining the advantages of HE with a dual-domain image decomposition algorithm. The proposed PSO algorithm is used to optimize the parameters to determine the enhanced image’s characteristics. Veluchamy et al. proposed a weighted gamma correction method based on the artificial bee colony (ABC) to improve the visual quality of contrast-distorted images [19]. First, image expansion and compression are individually applied to expose and restrict image intensity levels. Then, a weight method is used to add necessary details in dark regions. Finally, ABC is employed to calculate the optimal weight parameters to maintain brightness. Ashish et al. divided the input image into low and high exposure regions and controlled the degree of over-enhancement through clipping and optimal weighting processes before applying HE [20]. They use a krill herd (KH) optimization algorithm to enhance the contrast of dark areas. Finally, they integrate the two sub-histograms to produce an enhanced image while maintaining the naturalness and fine details of the original image.

In modern healthcare, precise diagnosis and treatment require accurate medical image segmentation. Saifullah et al. conducted a comprehensive study on the effectiveness of combining PSO with HE for image segmentation [21]. The optimal cost values reveal the strong performance of the PSO algorithm, while the HE preprocessing demonstrates significant stability, especially for complex lung CT scan images. Jiang et al. utilized the mathematical concepts of symmetric group theory to tackle the optimization challenge of image enhancement [22]. Their approach optimizes a hybrid objective function for multiple measurements in medical images and improves the contrast of intensity distribution. The enhanced ROI contributes to early diagnosis and patient survival rates, and it has a strong impact on the intermediate features and final results of computer-aided diagnosis (CAD) systems. Golabian et al. introduced a new medical image contrast and edge-sharpening enhancement framework based on the KH algorithm [23]. The approach trims the histogram using the minimum, maximum, mean, and median values with adjustable parameters. KH is used to optimize these parameters, and residual pixels are reallocated to the relatively empty bins in the histogram. Digital imaging technology has been widely applied in various fields. These technologies significantly improve the efficiency of medical image processing and ensure the accuracy of clinical diagnoses. Ye et al. investigated the WOA algorithm [24] to optimize the gamma correction parameters for image enhancement and presented the detailed visualization of medical images. Elnaz et al. applied the black hole algorithm and PSO (BHOPSO) to adjust the parameters of the mapping function for generating new pixel intensities [25]. The proposed framework overcomes the limitations of traditional enhancement techniques. Saifullah et al. proposed an improved U-Net image enhancement using PSO to address the complexity of brain tumor segmentation [26]. By employing the adaptive capabilities of PSO-based image enhancement, this technique achieves excellent performance on brain MRI images and enhances medical image analysis by providing accurate segmentation.

From the above literature, we can find that metaheuristic algorithms are superior to traditional algorithms in image enhancement. Still, they also have the problem of easily falling into local optima and often neglect to preserve fine details or edges in images. This paper proposes a method for enhancing the contrast of grayscale images based on the AWOA algorithm that utilizes its advantages of fast search speed and effective results. It maintains image details and suits for image enhancement tasks.

## 3. Proposed Image Enhancement Methodology

**Figure 1:**
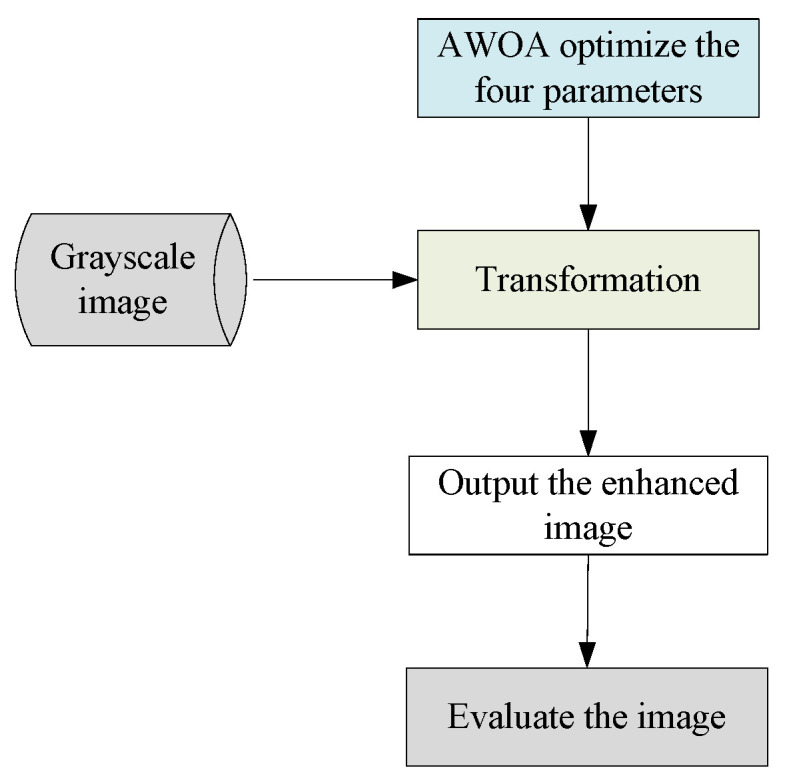
The process of image enhancement.

Image enhancement is accomplished by utilizing a transformation function, and AWOA fine-tunes its four parameters. The whole process is illustrated in Figure 1.

### 3.1. Objective Function

The transformation function in Equation (1) is utilized for image enhancement [18,27], which considers both global and local information.

(1) $$g\left(x,y\right)=k\times \frac{G}{\sigma (x,y)+b}\left[f\left(x,y\right)-c\times m\left(x,y\right)\right]+m{\left(x,y\right)}^{a}$$

where (x,y) represents a pixel of an image, and *f* and *g* mean the input and the enhanced images. *m* and σ are the local mean and standard deviation over an n×n window, as depicted in Equations (2) and (3).

(2) $$m\left(x,y\right)=\frac{1}{n\times n}\sum_{x=0}^{n-1}\sum_{y=0}^{n-1}f\left(x,y\right)$$

(3) $$\sigma \left(x,y\right)=\sqrt{\frac{1}{n\times n}\sum_{x=0}^{n-1}\sum_{y=0}^{n-1}{\left[f\left(x,y\right)-m\left(x,y\right)\right]}^{2}}$$

The global mean (*G*) of the input image is given by:

(4) $$G=\frac{1}{M\times N}\sum_{x=0}^{M-1}\sum_{y=0}^{N-1}f\left(x,y\right)$$

where *M* and *N* are the size of the input image.

The transformation function enhances image contrast by using the local mean as the enhancement center. The parameter *a* adjusts the brightness and smoothness of an image, while *b* introduces a shift based on the standard deviation of the surrounding pixels. *c* determines the fraction of the mean value subtracted from f(x,y), and *k* controls global enhancement. These four parameters significantly influence the processed image, so they need to be set precisely. The proposed AWOA must find the optimal combination to provide the best enhancement for the given images. According to [18,27], a∈[0,1.5], b∈[0,0.5], c∈[0,1], and k∈[0.5,1.5], respectively.

An objective function is necessary to evaluate the quality of the enhanced images without human intervention. A well contrast-enhanced image should have high-intensity edges, a large number of edges (edge pixels), and a high entropy value. We use Equation (5) as the objective function [18].

(5) $$F\left(g\right)=log\left(log\left(E\left(g\right)\right)\right)\times \frac{edgels(g)}{M\times N}\times e^{H(g)}$$

where edgels(g) represents the number of edge pixels in the enhanced image obtained through the Sobel edge detection algorithm. E(g) is the sum of pixel intensities in the M×N enhanced image, and H(g) is the entropy of the enhanced image.

### 3.2. Advanced Whale Optimization Algorithm

The WOA is a metaheuristic optimization algorithm based on whale hunting behavior [28]. Whales randomly choose between spiral motion and circling their prey. The update equation for spiral motion is as follows:

(6) $$X_{i}\left(t+1\right)=\left|X^{*}\left(t\right)-X\left(t\right)\right|\cdot e^{l}\cdot \cos\left(2\pi l\right)+{X_{i}}^{*}\left(t\right)$$

where $X_{i}\left(t+1\right)$ represents the position of *i* at t+1 iteration, and $X^{*}$ represents the global optimal solution.

(7) l=(a2−1)×rand()+1

where a2 decreases linearly from −1 to −2.

When circling prey, the search direction is determined by the value of *A*.

(8) $$X_{i}\left(t+1\right)=\left\{\begin{array}{c} X^{*}\left(t\right)-A\cdot |2\times rand\left(\right)\times X^{*}\left(t\right)-X_{i}\left(t\right)|\;if(|A|\geq 1)\\ X_{r}-A\cdot \left|2\times rand\left(\right)\times X_{r}\left(t\right)-X_{i}\left(t\right)\right|\;\;else \end{array}\right.$$

where $X_{r}$ represents a random solution.

(9) A=2a·rand()−a

where *a* decreases linearly from 2 to 0, and rand*() is a random function.

1. New exemplars

$X_{r}$ is a solution randomly selected from the population during the random search phase in WOA. This method forces $X_{i}$ to leave its prey and move towards $X_{r}$. It enhances the algorithm’s exploration ability and reduces the probability of falling into a local optimum. If $X_{r}$ is an individual with a higher objective function value in the population, there is a higher probability of generating a superior individual when $X_{i}$’s position is updated. If $X_{r}$ directly selects the best solution in the population, WOA’s global search ability is the weakest. On the contrary, WOA’s local search ability is at its weakest when $X_{r}$ selects the worst solution. Therefore, by choosing solutions with better objective function values as $X_{r}$ for position updates, $X_{i}$ can be closer to optimal solutions. In the proposed AWOA, we utilize only the top-performing half of the individuals as exemplars for the worst half to learn from. It not only increases the likelihood of producing excellent individuals after the position update during the search phase but also gives WOA a certain level of exploration capability.

2. Circling position update

WOA primarily relies on the fluctuation of the coefficient *A* to balance exploration and exploitation. When |A|≥1, the population is in the exploration phase, and WOA expands its search area to find better solutions. When |A|<1, the population enters the exploitation phase, and WOA focuses on searching around the position vector of the best solution.

**Figure 2:**
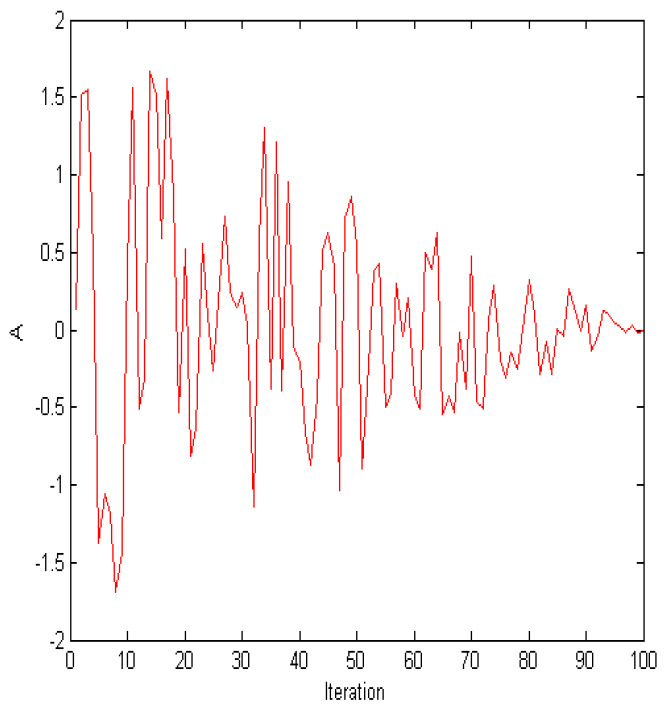
The values of A.

According to Equation (9), *A*∈ [−a, *a*]. When the value of *a* reaches 1 (at half of the maximum number of iterations), |A| remains less than 1, and WOA can no longer explore new solutions. Figure 2 depicts the value range of *A*. WOA balances exploration and exploitation during its early stages. To ensure that WOA still has global search capability in the later stages, AWOA improves Equation (8) as follows.

(10) $$X_{i}\left(t+1\right)=\left\{\begin{array}{c} X^{*}\left(t\right)-A\cdot \left|2\times rand\left(\right)\times X^{*}\left(t\right)-X_{i}\left(t\right)\right|\\ \;if(|A|\geq 1||(|A|<1\&rand()<t/MAX\_IT))\\ X_{rand}-A\cdot \left|2\times rand\left(\right)\times X_{rand}\left(t\right)-X_{i}\left(t\right)\right|\;\;else \end{array}\right.$$

where MAX_IT represents the maximum iteration. The new equation ensures that AWOA has global search capability even when |A| is less than 1.

3. Spiral position update

**Figure 3:**
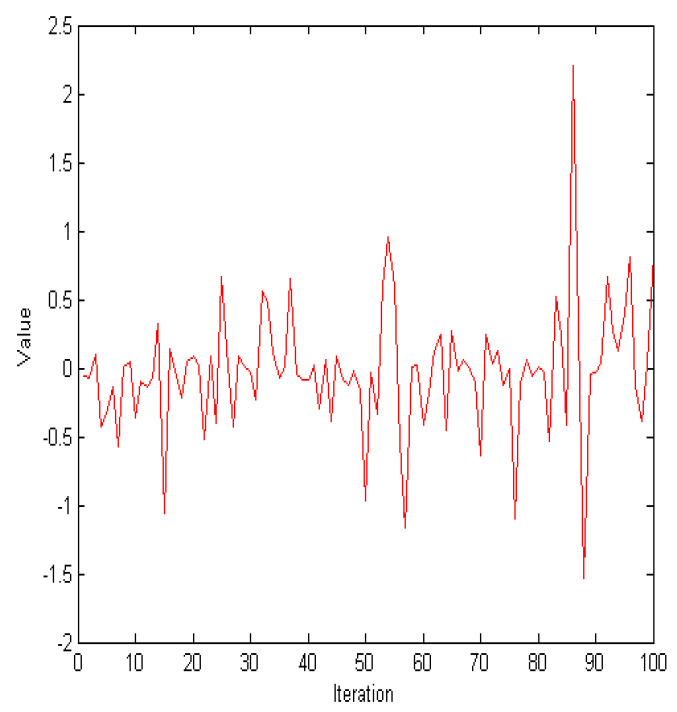
The values of $e^{l}\cdot \cos\left(2\pi l\right)$.

In WOA, the spiral update plays a key role in simulating the hunting behavior of whales. The diversity of solutions during the optimization process is increased by this spiral mechanism, which enhances the algorithm’s ability to escape local optima and improves global search effectiveness. During the spiral phase, the population searches for the global optimal solution. As illustrated in Figure 3, the term $e^{l}\cdot \cos\left(2\pi l\right)$ in Equation (6) does not exhibit a decreasing trend. To balance the global and local searches of the algorithm, the new update position for spiraling is defined as follows:

(11) $$X_{i}\left(t+1\right)\left\{\begin{array}{c} |X^{*}\left(t\right)-X\left(t\right)|\cdot e^{l}\cdot \cos\left(2\pi l\right)+{X_{i}}^{*}\left(t\right)\\ \;if(|A|\geq 1||(|A|<1\&rand()\geq t/MAX\_IT))\\ |X^{*}\left(t\right)-X\left(t\right)|\cdot e^{l}\cdot \cos\left(2\pi l\right)\cdot \left(1-t/MAX\_IT\right)+{X_{i}}^{*}\left(t\right)\;\;else \end{array}\right.$$

4. The whole process of AWOA

**Figure 4:**
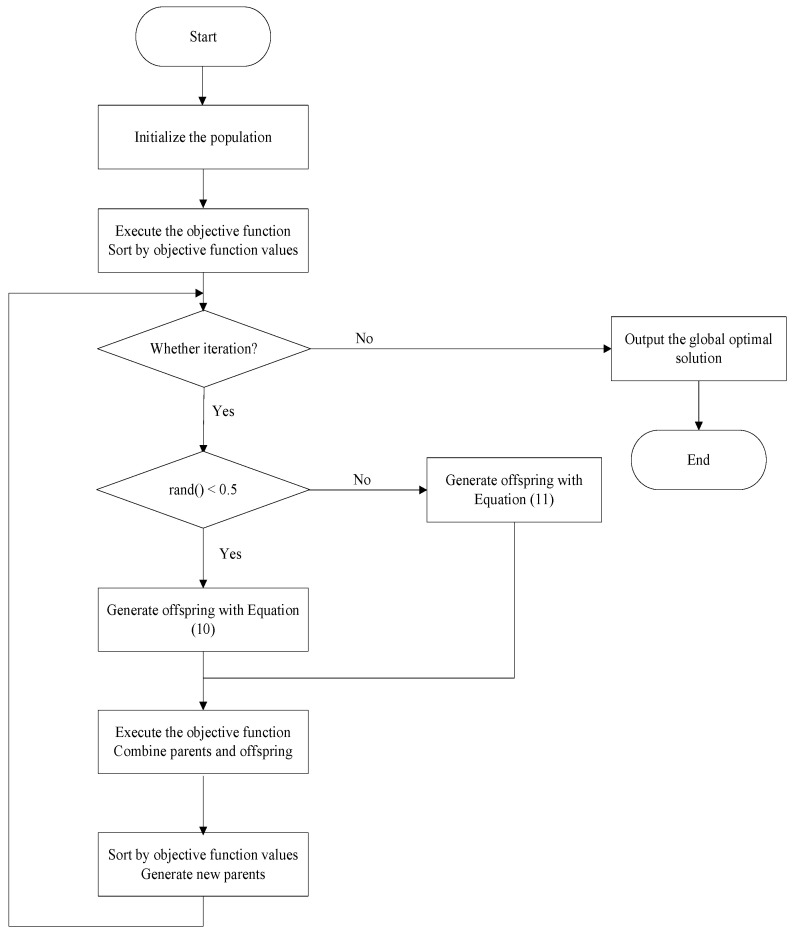
The process of AWOA.

If a new solution is superior to its parent solution, it will replace the parent solution in the next population. Otherwise, it is immediately discarded, and the parent solution will be retained. Figure 4 shows the process of AWOA, including circling position update and spiral update strategies through new exemplars.

The main factors that determine the time complexity of AWOA are the population size (*N*), the number of iterations (*T*), and the computation of the objective function (O(f)). In each iteration, the AWOA algorithm calculates the objective function value, while the computational cost of this process is typically proportional to the problem’s scale (*L*). Consequently, the overall time complexity is the sum of the objective function evaluation and position update O(T×N×f(L)+T×N×D), where *D* is the dimensionality of the AWOA algorithm.

## 4. Experimental Results and Analysis

**Figure 5:**
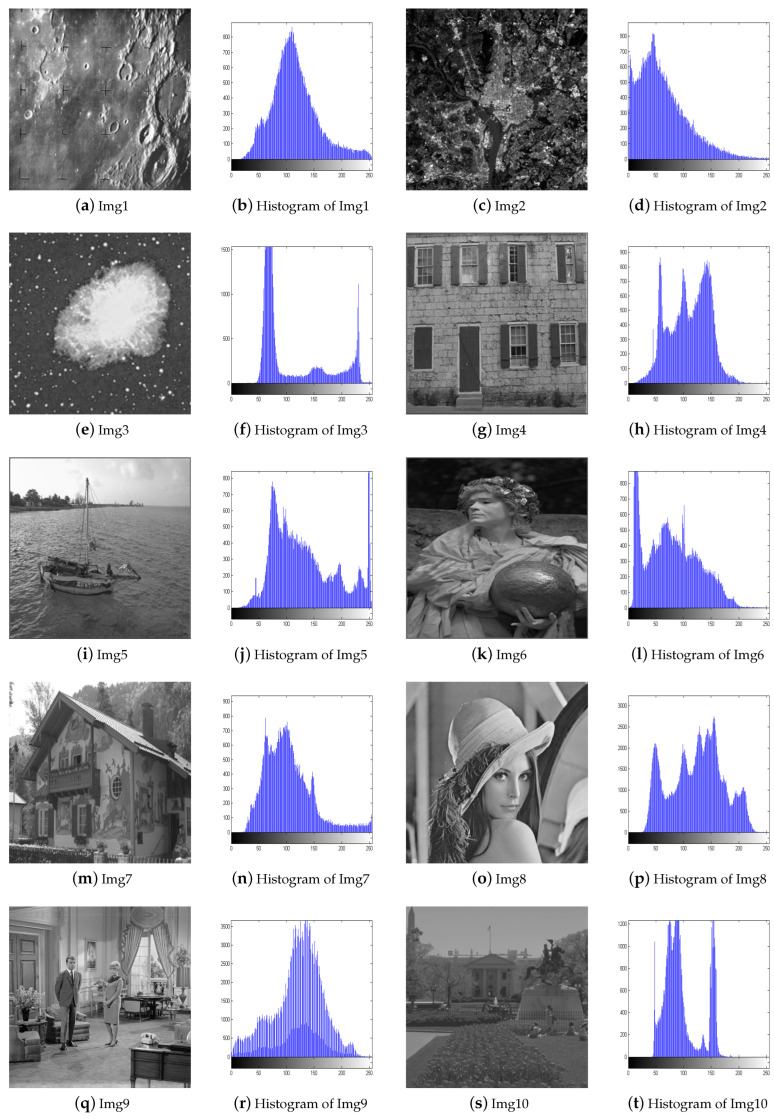
The test images and their histograms.

**Figure 6:**
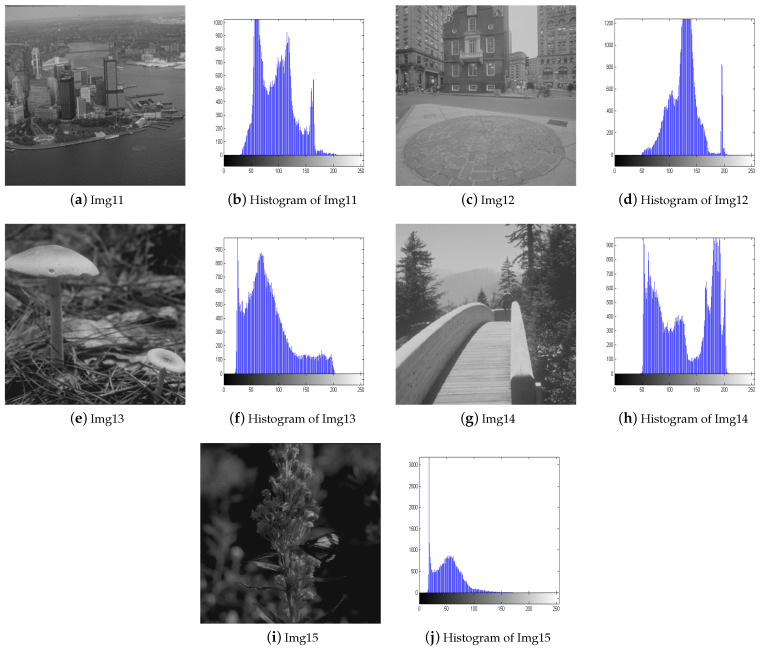
The test images and their histograms.

To validate the effectiveness of our proposed algorithm, we select 15 images from four test image sets [29,30,31] and compare them with four different image enhancement algorithms, including BHOPSO [25], ECS [18], PSO [26], and WOA [32]. For all algorithms, the population size is set to 20, and the maximum number of evaluations is set to 2000. To ensure a fair comparison, They use their default parameters. The test images include satellite, natural, benchmark, and low-contrast images. Except for Img8 and Img9, which are 512 × 512 pixels in size, the others are 256 × 256 pixels. Figure 5 and Figure 6 display the test images and their histograms.

### 4.1. Experimental Analysis

All the experimental data presented in this study are derived from the average results obtained after executing each algorithm 20 times. For the convenience of reading, the optimal solutions obtained by the algorithms in the experiment are displayed in bold font. Table 1 summarizes the objective function values of these algorithms in test images. AWOA outperforms the others algorithms, and it achieves the best objective function value in 11 out of the 15 test cases. This demonstrates AWOA’s robustness and effectiveness in enhancing image quality across a wide range of images. In contrast, ECS performs well in four images. BHOPSO consistently exhibits the poorest performance in the images tested.

It is particularly noteworthy that the algorithms perform exceptionally well in Img6 and Img9, where they show competitive results. However, the performance could be more favorable for Img13 and Img15, as the algorithms struggle to achieve significant enhancements. WOA outperforms the original WOA in all 15 test images.

To validate the statistical significance of the experimental results, the Wilcoxon rank sum test is applied with a significance level of 0.05. The test confirms that AWOA exhibits the best overall performance. ECS and AWOA demonstrate identical data distributions in Img6, Img10, and Img13, and AWOA excels in 14 images. AWOA outperforms all other algorithms in terms of performance ranking, followed by ECS, WOA, PSO, and BHOPSO. These findings underscore the effectiveness of the proposed AWOA algorithm in grayscale image enhancement tasks and highlight significant improvements over traditional optimization methods.

**Figure 7:**
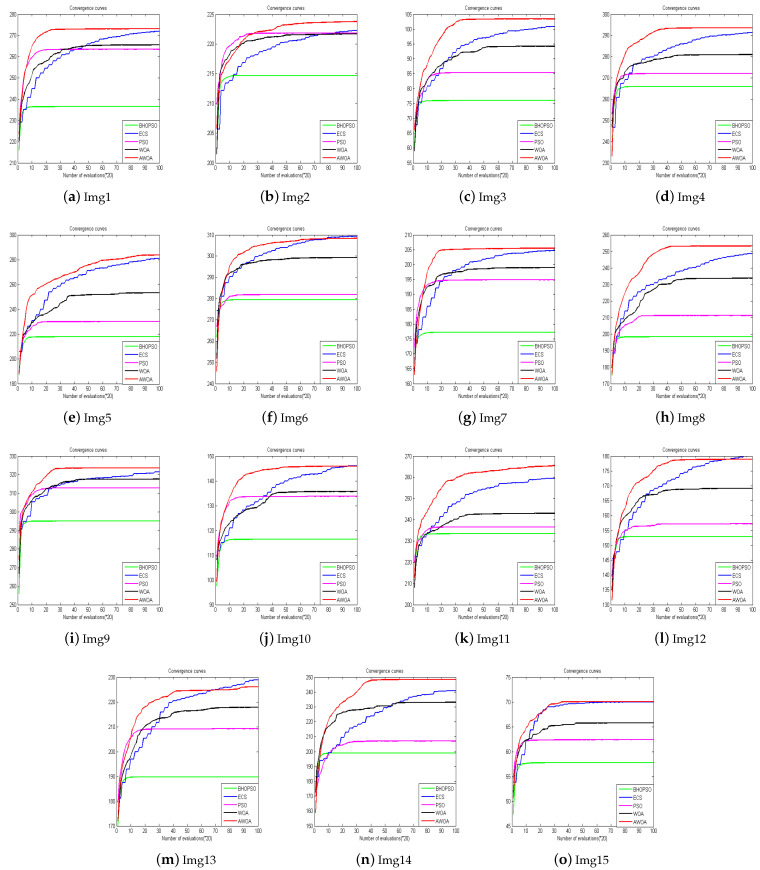
Convergence cures.

Figure 7 illustrates the convergence curves of the algorithms in 15 images. To facilitate display, the values on the x-axis in the figure are multiplied by 20 to reflect the actual evaluation numbers. It is evident that in Img1, Img3, Img4, Img8, Img9, Img11, and Img14, AWOA demonstrates superior convergence compared to the other algorithms, and it also obtains the best objective function values. In Img2 and Img7, although AWOA’s convergence is not as fast as PSO and WOA, it achieves better solutions through extensive exploration. For Img5, AWOA not only surpasses the other algorithms in terms of convergence and optimal solution but also maintains a continuously updated optimal solution throughout the process. In Img6, Img10, Img12, and Img13, while AWOA shows the best convergence, its final optimal solution is slightly worse than ECS. AWOA and ECS have very similar optimal solutions in Img15, but AWOA converges faster than ECS. AWOA is able to effectively explore the solution space and optimize the enhancement process more efficiently in these images.

Table 2 presents the computation time for different images. It is evident from the table that the proposed algorithm has a longer runtime compared to ECS, PSO, and WOA. PSO runs most efficiently, while WOA and AWOA have the longest running times. PSO’s logical structure is the simplest, and WOA’s structure is more complex. Both the algorithm’s logic and the objective function influence the runtime of image enhancement, as the objective function in this study is simple. The algorithms consume the least time in Img1–Img7 and Img10–Img15, while they take the most time in Img8 and Img9. This is because Img8 and Img9 are larger than the other images, and the execution time of the objective function is correlated with image size. Despite the increase in AWOA’s runtime, it remains within an acceptable range.

Table 3, Table 4, Table 5 and Table 6 present a comprehensive comparison of the five algorithms in terms of PSNR, FSIM, SSIM, and PCQI. These tables facilitate a deeper understanding of each algorithm’s effectiveness in enhancing various aspects of image quality.

When evaluated using the PSNR metric, AWOA performs slightly better than WOA. Specifically, AWOA achieves higher PSNR values than the other algorithms in images such as Img2, Img6, Img8, Img10, Img11, and Img15. However, in three images, Img3, Img5 and Img14, AWOA’s PSNR values are lower than those of the other algorithms. BHOPSO, ECS, and PSO achieve the highest PSNR values in 5, 2, and 2 images, respectively. AWOA is more effective at preserving the structural integrity of the images while simultaneously reducing noise and distortion.

From the results of Table 4, it is evident that AWOA significantly improves image contrast. AWOA achieves higher FSIM values compared to BHOPSO, ECS, PSO, and WOA in 11 images, including Img1, Img3, Img4, Img7, Img8, Img9, Img10, Img11, Img12, Img14, and Img15. AWOA is particularly effective at enhancing the overall detail and complexity of the images. On the other hand, BHOPSO and WOA perform relatively well in Img13 and Img2, since they achieve a higher FSIM value than the other algorithms. ECS outperforms the other algorithms in Img6 and Img7. Overall, AWOA outperforms BHOPSO, ECS, PSO, and WOA in terms of enhancing image detail and information content, and it is well-suited for applications where preserving fine image details and enhancing contrast are critical for achieving high-quality results.

In terms of SSIM, BHOPSO, ECS, WOA, and AWOA demonstrate varying levels of effectiveness across the test images. AWOA stands out, and it achieves the best results in 13 out of the 15 images. BHOPSO and PSO show no advantage in any of the tested images. The results of SSIM suggest that AWOA is particularly adept at preserving and enhancing the sharpness of edges.

PCQI is a non-reference image quality assessment method and it evaluates image quality based on local contrast and structural information. BHOPSO, ECS, PSO, WOA, and AWOA achieve the highest PCQI values in 1, 7, 0, 0, and 7 images, respectively. Although ECS and AWOA perform equally well, AWOA demonstrates excellent stability. It ranks second in Img1, Img4, Img5, Img6, Img10, Img12, and Img13. In contrast, ECS is extremely unstable, while it performs poorly in Img15. AWOA has the highest average ranking, followed by ECS, WOA, PSO, and BHOPSO. The algorithms significantly enhance the contrast of local patches in Img1–Img14, and they effectively improve the visibility of details and edges. However, the local contrast of Img15 is originally low.

In conclusion, based on the objective function, PSNR, FSIM, SSIM, and PCQI, AWOA outperforms BHOPSO, ECS, WOA, and PSO. Its superior performance in preserving image details, enhancing edges, and improving clarity makes it highly suitable for image enhancement tasks.

### 4.2. Evaluation of the Enhanced Images Visually

**Figure 8:**
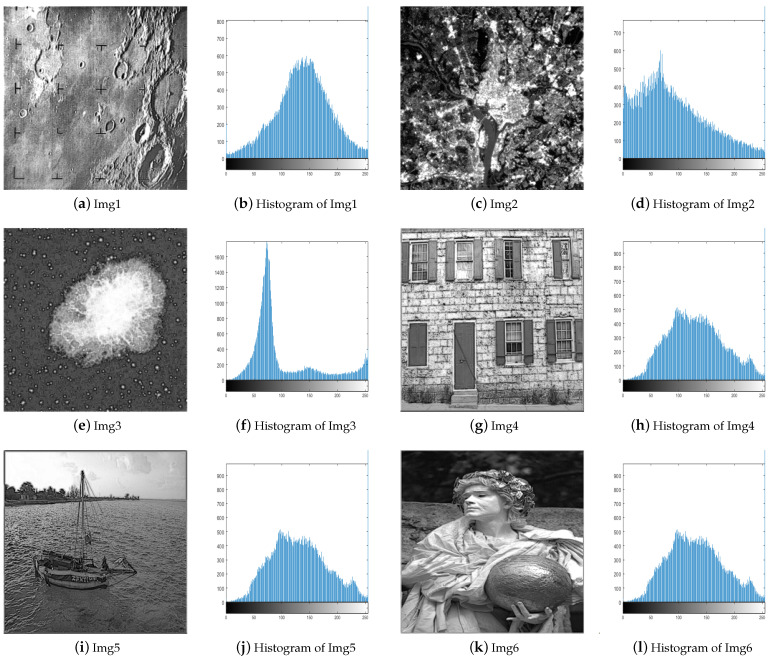
The enhanced images and their histograms.

**Figure 9:**
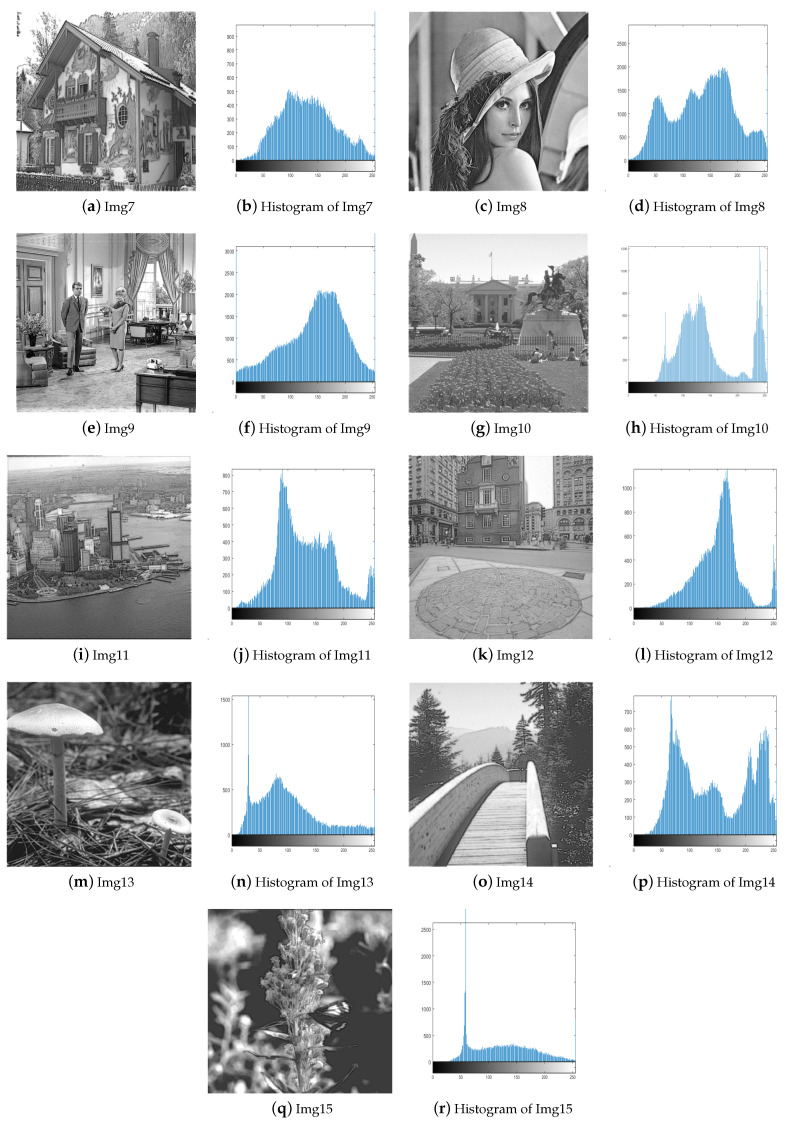
The enhanced images and their histograms.

In this section, we compare the efficiency of the proposed AWOA in improving the contrast and detail of images. Figure 8 and Figure 9 exhibits the enhanced images produced by AWOA.

Img1–Img3 are satellite images, and they all appear significantly clearer after enhancement. AWOA adjusts the brightness distribution of these images and brings out more details in the originally dark areas. The histograms of the enhanced images demonstrate a greater pixel distribution in the low brightness region, so their distribution is more balanced. The images for Img4–Img6 appear more natural after enhancement. AWOA reduces the multiple peaks in the original images, and the pixel value distribution is more uniform. The enhanced images not only improve contrast but also preserve important details, such as the door frame, water ripples, and folds in clothing. These are critical features for maintaining the authenticity and fine texture of the images. By applying contrast stretching to Img7 and Img8, the image details become more clearly visible. In the histogram of Img7, multiple small peaks can be seen, indicating that details at different brightness levels are more prominently highlighted. The original images of Img10-Img15 are set with different low illumination conditions and exhibit narrower histograms. The histograms of the enhanced images are more dispersed and demonstrate an even distribution. The clarity of these images is also enhanced.

The enhanced images produced by AWOA are visually closer to the original images, which is a strong indicator of the algorithm’s effectiveness in improving image clarity without introducing significant distortion. This visual similarity reflects AWOA’s ability to enhance image details while maintaining the natural appearance of the images.

### 4.3. Compared with Histogram Equalization Methods and Ablation Experiments

To evaluate the effectiveness of the proposed algorithm, we compare it with histogram equalization methods, such as HEF [33] and CAHE [34]. Table 7, Table 8, Table 9 and Table 10 present experimental data for these algorithms in terms of PSNR, FSIM, SSIM, and PCQI. AWOA demonstrates exceptional performance in PSNR, FSIM, and SSIM, and it outperforms the comparison algorithms in 13, 11, and 13 images, respectively. AWOA is highly effective in preserving structural integrity. However, AWOA performs poorly in PCQI, falling behind CAHE and HEF. AWOA proves to be an ideal choice for tasks where global image enhancement and overall clarity are the primary concerns, but further refinement may be needed for applications that heavily rely on local contrast preservation.

We improve the AWOA algorithm from new exemplars, circling position update, and spiral update. As shown in Section 4.1, they have excellent image enhancement effects. Table 11 presents the results of ablation experiments conducted to analyze the impact of these improvements on the algorithm. The experimental data shows the number of instances in which the proposed improvement strategy outperforms WOA. The three improvement measures significantly improve the performance of the WOA algorithm, especially new exemplars and circling position update. These measures are effective and can be applied in image enhancement.

## 5. Conclusions

This paper presents an advanced WOA algorithm for grayscale image enhancement. The objective function in this study takes into account the entropy and the number of edge pixels in images. The algorithm is implemented using a transformation function that contains both global and local information. The proposed AWOA is used to optimize the four key parameters of this function. New exemplars and position updates are utilized in the algorithm. The proposed algorithm is compared with other algorithms, and the results indicate its superiority in subjective qualitative and objective quantitative evaluations.

While the proposed AWOA demonstrates excellent performance in enhancing grayscale images, its applicability to color and high-resolution images remains underexplored. Grayscale images involve a single channel, which simplifies the optimization process. However, color images consist of multiple channels (R, G, B), and the independent or joint optimization of parameters for each channel introduces additional complexity. High-resolution images significantly expand the search space, and they lead to increased computational costs and longer processing times. Although AWOA has shown effective convergence for smaller images, it may be difficult in real-time applications. As with metaheuristic algorithms, AWOA is sensitive to parameter settings such as population size and iteration count. Improper tuning of these parameters can cause premature convergence or slow optimization. We can implement an adaptive mechanism to dynamically adjust these parameters based on the characteristics of the image content or the search space. Additionally, parallelization strategies could accelerate AWOA for large-scale or real-time applications, especially for high-resolution images. Satellite and medical imaging can be challenging to interpret for land monitoring and disease diagnosis due to noise, low contrast, and low resolution. The proposed AWOA enhances image contrast and clarity, and significantly aids diagnostic and analytical processes.

## Figures and Tables

**Table 1:** The objective function values of the metaheuristic algorithms.

Image	BHOPSO	ECS	PSO	WOA	AWOA
Img1	236.4640	271.8410	263.4538	265.5736	**273.1474**
Img2	214.6902	222.2742	221.8189	221.7011	**223.7718**
Img3	75.9251	100.8623	85.3670	94.2189	**103.4280**
Img4	265.9714	291.2755	272.0849	280.9921	**293.6178**
Img5	217.5964	280.8553	229.9027	253.2028	**283.7132**
Img6	279.4530	**309.2412**	281.8684	299.2884	308.4039
Img7	177.2104	204.6594	194.8185	198.9765	**205.5083**
Img8	198.2655	248.6043	211.0919	233.7106	**253.2086**
Img9	295.0253	321.4752	312.8841	317.6043	**323.6732**
Img10	116.3791	**146.1243**	133.7074	135.7148	145.9548
Img11	233.3677	259.5309	236.5904	243.0281	**265.5205**
Img12	152.9294	**179.9783**	157.2019	169.1388	178.9208
Img13	189.8207	**228.7925**	209.1573	217.8172	226.0824
Img14	199.0663	240.9255	207.1201	233.0510	**248.4387**
Img15	57.7229	69.9964	62.4156	65.7773	**70.0766**
>/=/<	0/0/15	4/0/11	0/0/15	0/0/15	11/3/1

The optimal solutions obtained by the algorithms in the experiment are displayed in bold font.

**Table 2:** The computation time of the metaheuristic algorithms.

Image	BHOPSO	ECS	PSO	WOA	AWOA
Img1	8.0031	6.0798	**5.0095**	6.9832	7.8374
Img2	8.0048	6.3238	**8.2197**	8.1975	8.2832
Img3	7.9764	6.1464	**4.7609**	6.7607	7.6379
Img4	8.0051	6.3083	**4.7807**	7.3282	7.8708
Img5	8.0300	6.2834	**6.3937**	7.9977	8.1644
Img6	8.0452	6.2520	**6.6992**	8.0391	7.9950
Img7	7.9986	5.8793	**4.8721**	7.0644	7.8576
Img8	37.7047	26.1667	**24.2211**	33.9242	35.2406
Img9	38.8838	26.0615	**24.1822**	35.7971	37.2330
Img10	8.0172	6.5635	**5.2292**	7.5356	8.0088
Img11	8.0561	6.1422	**5.0081**	7.5765	8.1614
Img12	8.0928	6.1102	**5.1043**	6.8948	7.8753
Img13	8.0397	6.3173	**6.8706**	8.3598	8.2271
Img14	8.0074	5.9832	**4.8297**	7.1798	7.7716
Img15	8.0460	6.1010	**7.9190**	6.9185	7.7554

The optimal solutions obtained by the algorithms in the experiment are displayed in bold font.

**Table 3:** The PSNR values of the metaheuristic algorithms.

Image	BHOPSO	ECS	PSO	WOA	AWOA
Img1	**17.6846**	17.2311	16.8940	17.2716	17.1458
Img2	14.2454	14.7904	14.4572	14.5135	**15.4639**
Img3	**26.0344**	23.9569	25.2053	24.1994	23.7172
Img4	13.5535	**14.2838**	13.5501	13.7586	14.2322
Img5	27.5348	23.2648	**29.5161**	26.2994	22.1189
Img6	15.6696	16.2233	15.6395	15.6746	**16.3080**
Img7	**17.6698**	16.2379	16.3127	15.8952	16.3991
Img8	21.4395	21.7143	21.4533	21.5859	**21.9276**
Img9	18.0139	17.9874	**18.0390**	18.0071	17.9884
Img10	16.7751	16.6088	16.4733	16.7555	**17.1117**
Img11	16.0860	16.6894	16.1905	16.3919	**16.9379**
Img12	15.9886	**19.2835**	16.1691	17.7842	19.1157
Img13	**21.9457**	20.7998	21.2461	20.9097	21.1201
Img14	**20.9835**	20.2605	20.6223	20.4291	20.1715
Img15	10.3853	10.7004	10.2366	10.7808	**10.7996**

The optimal solutions obtained by the algorithms in the experiment are displayed in bold font.

**Table 4:** The FSIM values of the metaheuristic algorithms.

Image	BHOPSO	ECS	PSO	WOA	AWOA
Img1	0.8363	0.8275	0.8409	0.8263	**0.8943**
Img2	0.8906	0.8952	0.8879	**0.9079**	0.9033
Img3	0.8314	0.8270	0.8583	0.8217	**0.8832**
Img4	0.7948	0.7603	0.8224	0.7562	**0.8327**
Img5	0.9002	**0.8561**	0.9354	0.8439	0.9280
Img6	0.8536	**0.8718**	0.8841	0.8569	0.8783
Img7	0.8032	0.7954	0.8132	0.7960	**0.8558**
Img8	0.9384	0.9270	0.9525	0.9220	**0.9561**
Img9	0.8828	0.8749	0.8912	0.8722	**0.9150**
Img10	0.8809	0.8681	0.8895	0.8540	**0.9110**
Img11	0.8543	0.8203	0.8663	0.8079	**0.8760**
Img12	0.8230	0.8091	0.8479	0.8047	**0.8666**
Img13	**0.9136**	0.9133	0.9078	0.8963	0.9081
Img14	0.8605	0.8498	0.8924	0.8433	**0.8958**
Img15	0.7587	0.7596	0.7488	0.7609	**0.7691**

The optimal solutions obtained by the algorithms in the experiment are displayed in bold font.

**Table 5:** The SSIM values of the metaheuristic algorithms.

Image	BHOPSO	ECS	PSO	WOA	AWOA
Img1	0.7591	0.7459	0.7670	0.7439	**0.8515**
Img2	0.8224	0.8300	0.8192	**0.8451**	0.8034
Img3	0.7826	0.7664	0.8263	0.7573	**0.8691**
Img4	0.7326	0.6948	0.7640	0.6906	**0.7751**
Img5	0.8827	0.8175	0.9297	0.8063	**0.9362**
Img6	0.7822	0.8016	**0.8209**	0.7899	0.8144
Img7	0.7389	0.7288	0.7547	0.7307	**0.8129**
Img8	0.8489	0.8146	0.8948	0.8085	**0.9165**
Img9	0.7808	0.7613	0.7991	0.7541	**0.8627**
Img10	0.8365	0.8161	0.8423	0.8104	**0.8760**
Img11	0.8110	0.7662	0.8239	0.7549	**0.8338**
Img12	0.7743	0.7552	0.8044	0.7536	**0.8266**
Img13	0.8908	0.8859	0.8894	0.8736	**0.8972**
Img14	0.8396	0.8255	0.8777	0.8171	**0.8899**
Img15	0.5718	0.5353	0.5794	0.5382	**0.6237**

The optimal solutions obtained by the algorithms in the experiment are displayed in bold font.

**Table 6:** The PCQI values of the metaheuristic algorithms.

Image	BHOPSO	ECS	PSO	WOA	AWOA
Img1	0.9629	**1.0109**	0.9905	0.9975	1.0104
Img2	0.9563	1.0029	0.9834	0.9774	**1.0177**
Img3	0.9429	1.0195	0.9656	0.9667	**1.0255**
Img4	0.9539	**1.0467**	0.9634	0.9921	1.0449
Img5	1.0642	**1.1876**	1.0993	1.1422	1.1678
Img6	1.0004	**1.0924**	1.0160	1.0458	1.0835
Img7	0.9681	1.0221	0.9733	0.9841	**1.0251**
Img8	1.0304	1.1250	1.0610	1.0997	**1.1272**
Img9	1.0206	1.1107	1.0789	1.0949	**1.1175**
Img10	0.9642	**1.0564**	1.0143	1.0215	1.0430
Img11	0.9754	1.0529	0.9780	1.0006	**1.0545**
Img12	0.9725	**1.1135**	0.9491	1.0196	1.0842
Img13	1.0030	**1.0780**	1.0384	1.0537	1.0580
Img14	0.9484	1.0099	0.9579	0.9991	**1.0175**
Img15	**0.9699**	0.9442	0.9394	0.9504	0.9455

The optimal solutions obtained by the algorithms in the experiment are displayed in bold font.

**Table 7:** The PSNR values of the AWOA and HE algorithms.

Image	AWOA	HEF	CAHE
Img1	**17.1458**	16.3111	15.7677
Img2	**15.4639**	10.6675	10.7976
Img3	**23.7172**	14.5628	12.8940
Img4	14.2322	**15.5402**	15.2576
Img5	**22.1189**	21.2190	12.7669
Img6	**16.3080**	12.9614	11.9429
Img7	**16.3991**	16.3233	14.0049
Img8	**21.9276**	19.4568	15.3155
Img9	**17.9884**	17.5546	15.0415
Img10	**17.1117**	16.2094	11.1434
Img11	**16.9379**	15.4838	13.1020
Img12	**19.1157**	16.6890	15.0781
Img13	**21.1201**	13.3802	12.9426
Img14	20.1715	**23.5663**	12.3956
Img15	**10.7996**	8.3767	10.0881

The optimal solutions obtained by the algorithms in the experiment are displayed in bold font.

**Table 8:** The FSIM values of the AWOA and HE algorithms.

Image	AWOA	HEF	CAHE
Img1	**0.8943**	0.8114	0.7683
Img2	**0.9033**	0.8161	0.7878
Img3	**0.8832**	0.5600	0.5105
Img4	**0.8327**	0.8242	0.7572
Img5	**0.9280**	0.8972	0.6592
Img6	0.8783	**0.9083**	0.7294
Img7	**0.8558**	0.8333	0.7789
Img8	**0.9561**	0.9326	0.7027
Img9	**0.9150**	0.8651	0.7592
Img10	**0.9110**	0.8608	0.7341
Img11	0.8760	**0.9021**	0.7598
Img12	**0.8666**	0.8099	0.7287
Img13	**0.9081**	0.8707	0.8384
Img14	0.8958	**0.9519**	0.7724
Img15	0.7691	**0.8891**	0.7941

The optimal solutions obtained by the algorithms in the experiment are displayed in bold font.

**Table 9:** The SSIM values of the AWOA and HE algorithms.

Image	AWOA	HEF	CAHE
Img1	**0.8515**	0.7296	0.6630
Img2	**0.8034**	0.6215	0.6031
Img3	**0.8691**	0.4723	0.3968
Img4	**0.7751**	0.7372	0.6660
Img5	**0.9362**	0.8535	0.5247
Img6	**0.8144**	0.7868	0.5889
Img7	**0.8129**	0.7427	0.6726
Img8	**0.9165**	0.8740	0.5668
Img9	**0.8627**	0.7827	0.6068
Img10	**0.8760**	0.7904	0.5575
Img11	0.8338	**0.8446**	0.5915
Img12	**0.8266**	0.7052	0.6003
Img13	**0.8972**	0.7353	0.7028
Img14	0.8899	**0.9212**	0.6045
Img15	**0.6237**	0.3893	0.5545

The optimal solutions obtained by the algorithms in the experiment are displayed in bold font.

**Table 10:** The PCQI values of the AWOA and HE algorithms.

Image	AWOA	HEF	CAHE
Img1	1.0104	1.1794	**1.2407**
Img2	1.0177	0.9843	**1.0416**
Img3	1.0255	1.1564	**1.2380**
Img4	1.0449	1.2439	**1.2752**
Img5	1.1678	1.1158	**1.2990**
Img6	1.0835	1.0266	**1.1974**
Img7	1.0251	1.1507	**1.2155**
Img8	1.1272	1.1139	**1.2900**
Img9	1.1175	1.1292	**1.2949**
Img10	1.0430	1.0613	**1.0902**
Img11	1.0545	1.0642	**1.2067**
Img12	1.0842	1.1750	**1.2764**
Img13	1.0580	0.9548	**1.0663**
Img14	1.0175	0.9429	**1.0208**
Img15	**0.9455**	0.7425	0.9452

The optimal solutions obtained by the algorithms in the experiment are displayed in bold font.

**Table 11:** The results of ablation studies.

Improvement	PSNR	FSIM	SSIM	PCQI
New exemplars	8	11	12	12
Circling position update	7	12	13	12
Spiral update	5	9	10	10

The optimal solutions obtained by the algorithms in the experiment are displayed in bold font.

## Data Availability

Data is contained within the article, further inquiries can be directed to the corresponding author.
